# Appendages of the Cyanobacterial Cell

**DOI:** 10.3390/life5010700

**Published:** 2015-03-04

**Authors:** Nils Schuergers, Annegret Wilde

**Affiliations:** University of Freiburg, Institute of Biology III, Schänzlestr. 1, 79104 Freiburg, Germany; E-Mail: Nils.Schuergers@biologie.uni-freiburg.de

**Keywords:** type IV pili, motility, surface structure

## Abstract

Extracellular non-flagellar appendages, called pili or fimbriae, are widespread in gram-negative bacteria. They are involved in many different functions, including motility, adhesion, biofilm formation, and uptake of DNA. Sequencing data for a large number of cyanobacterial genomes revealed that most of them contain genes for pili synthesis. However, only for a very few cyanobacteria structure and function of these appendages have been analyzed. Here, we review the structure and function of type IV pili in *Synechocystis* sp. PCC 6803 and analyze the distribution of type IV pili associated genes in other cyanobacteria. Further, we discuss the role of the RNA-chaperone Hfq in pilus function and the presence of genes for the chaperone-usher pathway of pilus assembly in cyanobacteria.

## 1. Introduction

Surface appendages are widespread among gram-negative bacteria, but have been found also on the surface of gram-positive bacteria. These structures can be observed by transmission electron microscopy coupled with negative staining and also by scanning electron microscopy. From the early days of bacterial genetics conjugative pili have attracted a lot of attention. Though, the most prominent extension beyond the surface of a bacterial cell is most probably the flagellum. Many bacteria use flagella for movement, but they are also important factors for virulence and adhesion [1,2]. On the other hand several bacteria are able to move across surfaces using non-flagellar motility machineries, such as type IV pili. In addition, most gram-negative bacteria have short, thin appendages, which are not involved in motility, but rather facilitate adherence to surfaces or other cells. These structures are not mutually exclusive and some bacteria synthesize a range of different surface appendages [3].

The gram-negative cyanobacteria do not contain genes encoding flagella components. Examination of cyanobacteria by electron microscopy revealed the existence of surface appendages in several unicellular cyanobacteria [4]. The most advanced studies on the function of these structures have been published for the model cyanobacterium *Synechocystis* sp. PCC 6803 (hereafter *Synechocystis* 6803). Molecular approaches support the role of type IV pili in motility and natural competence [5,6,7]. As most cyanobacteria harbor genes encoding proteins homologous to those of the type IV pilus apparatus, we are convinced that type IV pili or alternative structures encoded by these genes have important functions in many other cyanobacteria. Based on a brief overview of the structure, function and regulation of type IV pili biogenesis in *Synechocystis* 6803, we discuss the potential presence of similar structures in other cyanobacteria and hint at specific differences in comparison to other gram-negative bacteria. 

## 2. Appendages of Bacteria: Classification and Nomenclature

According to Fronzes *et al.* [8], non-flagellar appendages of gram-negative bacteria are classified into five major groups: Conjugative pili (homologous to the type IV secretion system), type IV pili, type III secretion needle/pili, curli and chaperone-usher (CU) pili (also known as type I pili). Type IV secretion pili are well known from bacteria that are able to transfer genetic material by conjugation. They establish a close contact from the host bacterium to other cells, even to eukaryotic cells. Some pathogenic bacteria use these appendages to transfer virulence factors, including DNA (*Agrobacterium tumefaciens*) or proteins (*Helicobacter pylori*). The size of type IV secretion pili varies from <1–20 μm depending on the specific system. Electron cryo-microscopy studies showed that the F-pilus has a central lumen of 3 nm in diameter that is theoretically large enough to allow for the passage of single-stranded DNA [9]. Transfer of the genetic material through the pilus lumen has been proposed, but never demonstrated [10,11].

**Figure 1:**
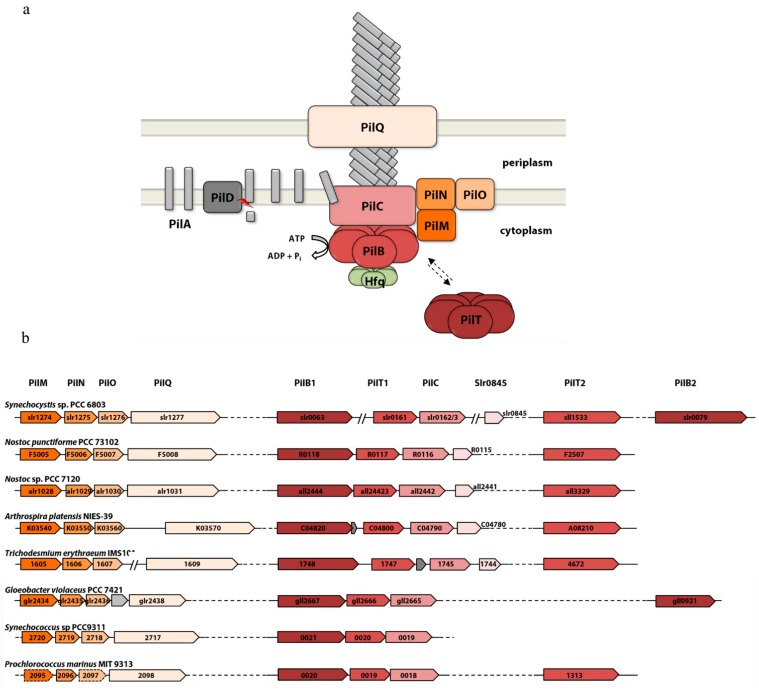
(**a**) Model of type IV pili and their assembly machineries in cyanobacteria. Most cyanobacteria harbor more than one PilA homolog. Their specific functions are unknown so far. (**b**) Synteny of *Synechocystis* 6803 gene clusters encoding type IV pili proteins in comparison with similar genes clusters of diverse cyanobacteria. Color-coding of open reading frames refers to the schematic view in (a).

Type IV pili are 6–8 nm in diameter and several μm in length and can form bundles. They are essential for many different processes like biofilm formation, aggregation, twitching motility and virulence [12,13]. Genes that encode core components of the type IV pilus show high sequence similarity, even if they originate from evolutionary divergent bacterial groups [14]. Type IV pili are assembled by a complex machinery at the inner membrane [15]. The main structural component of the pilus (PilA) is produced as a precursor. PilD removes the N-terminal leader sequence and methylates the protein. The assembly of the pilus requires a large ATP driven complex. This machinery is also essential for the reversibility of assembly and retraction of the pilus, a major feature of type IV pili. The PilB ATPase is required for pilus assembly, whereas PilT energizes depolymerisation of the pilus. Both ATPases are located at the base of the pilus and most probably interact with PilC, which is embedded in the inner membrane. PilQ forms the pore for the pilus to cross the outer membrane (Figure 1a). According to differences in their subunits, assembly system, and gene organization, two type IV pili subfamilies can be distinguished. Type IVa pili are widely distributed and highly conserved among many bacterial phyla and typically associated with twitching motility. The more heterogeneous type IVb pili are only found in a small subset of genera and often lack an ATPase for pilus retraction [14].

Type III secretion needles are mostly known from pathogenic bacteria like *Salmonella typhimurium*. They use this, so called injectisome, to secrete effector proteins into the eukaryotic cell thereby assisting colonization of the host cell. This short, rigid structure is approximately 60 nm long and has an inner diameter of 2–3 nm. Assembly of the type III secretion system shares similarities to flagella assembly [16].

Curli have been described as abundant amyloid extracellular fibers of 6–10 nm produced by many Enterobacteria (reviewed in [17]). These protein structures are the major component of *E. coli* biofilms and contribute to the adhesion of pathogenic bacteria to cells.

CU pili are found in many bacteria and it seems that they form the most abundant group of bacterial surface appendages [8]. The surface structures assembled by the chaperon-usher pathway can differ in their morphology and function and may even form non-fimbrial structures. Based on operon structure, phylogeny and morphology, CU pili are classified into α-, β-, γ-, κ-, π- and σ-fimbriae [18]. CU operons contain at least one major structural subunit, a periplasmic chaperon and a membrane protein for translocation of the subunits (the so called usher protein) [18]. The Sec translocase is involved in transport of the subunits across the inner membrane, which are then folded and stabilized with the help of the chaperone in the periplasm. After binding to the usher protein, which forms a pore in the outer membrane, the chaperone is displaced and the pilin is integrated into the filament on the surface. Further details are discussed in Fronzes *et al.* [8].

### Appendages of Cyanobacteria

Vaara [4] demonstrated the presence of pili in 11 out of 22 analyzed unicellular cyanobacterial strains. The author showed that these surface structures are not specific for cyanobacteria and resemble those of other bacteria. However, in contrast to well-characterized appendages of model *Proteobacteria*, these structures are largely genetically and biochemically uncharacterized. Moreover, appendages are referred to in different studies as pilus-like structures [19], fibers [20] or spinae [21] resulting in terminological confusion. Without detailed analysis of these components, it is not possible to evaluate whether we deal with different structures described in these studies.

In *Synechocystis* 6803, two different types of pili have been defined using negative staining [6,7]. One morphotype is defined by an average diameter of 3–4 nm and a length of approximately 1 μm. These thin pili cover the whole surface of the cell. The second morphotype is specified as thick and long pili with a diameter of 6–8 nm and more than 2 μm in length. The thick pili often appear as tuffs and have been identified as type IVa pili by genetic methods [5,6,7]. 

There is also evidence that hormogonia developed by the symbiotic cyanobacterium *Nostoc punctiforme* possess pilus-like structures all over the surface, whereas the vegetative cells of the parent filaments lack pili [22,23]. However, by using antibodies against the putative major pilin PilA Risser *et al.* [24] demonstrated that this subunit accumulated exclusively in rings at the junction between cells. The authors suggest that this putative pilin is part of the previously described junctional pore complex involved in motility [25].

Transmission electron microscope studies showed pilus-like structures on the surface of *Microcystis aeruginosa* PCC 7806 cells [26]. Appendages from cells grown in liquid culture were comparable to the thick pili shown by Bhaya *et al.* [6]. In contrast, when cells from agar plates were analyzed, they exhibited thicker pili with diameters of 20 to 35 nm, which may consist of bundles of thinner filaments [26]. Thus, growth conditions may influence composition and morphology of appendages. Homologs to type IV pili genes have been identified in this organism.

## 3. Type IV Pili of Cyanobacteria

The type IV pili apparatus is a complex, multi-protein machinery conserved in a wide variety of bacteria. Phylogenetic analyses suggest that several of its evolutionary ancient protein constituents are homologous to those of the type II secretion system and the archaellum [27,28]. The function of most cyanobacterial type IV pili proteins was not investigated on a biochemical and structural level in detail. Nevertheless, their role in pilus assembly can be inferred from mutant phenotypes observed in *Synechocystis* 6803 and *Nostoc punctiforme* and their similarity to the widely studied homologs from gram-negative model organisms like *Neisseria*, *Pseudomonas* or *Myxococcus xanthus* (reviewed in [15,29]). The type IV pili apparatus is composed of four distinct subcomplexes that together span the inner and outer membranes: The pilus rod, the outer membrane complex, the cytoplasmic pilus platform and the secretion ATPases (see Figure 1a). The genes encoding components of the cyanobacterial type IV pili apparatus were originally identified in *Synechocystis* 6803*.* Figure 1b shows gene organization of operons and orphan genes in the genome of *Synechocystis* 6803 in comparison to other cyanobacteria.

### 3.1. The Membrane Complexes

Not much is known about the membrane components of the cyanobacterial type IV pili apparatus. PilQ is a secretin family protein that forms a homo-multimeric pore complex, which facilitates the transport of the pilus subunits across the outer membrane. PilMNO proteins are thought to be important for aligning the pore complex with the pilus platform [30,31,32]. Genes encoding these membrane proteins are conserved within a putative operon structure in most cyanobacteria (Figure 1b). In *Synechocystis* 6803 disruption of these genes leads to loss of type IV pili, motility and natural competence, while cells retain (bundles) of thin pili [7]. Remarkably, we were not able to identify PilP homologs in cyanobacteria, although this protein is encoded in the highly conserved *pilMNOPQ* gene cluster in essentially all other gram-negative bacteria harboring type IVa pili genes. The lipoprotein PilP is thought to connect the PilQ secretin pore with the inner membrane proteins PilN and PilO, thereby bridging the periplasm [33]. Despite their overall gram-negative structure, the peptidoglycan layer of cyanobacteria is substantially thicker than that of most gram-negative bacteria [34]. Hence, we speculate that the periplasmic structure of the alignment complex in cyanobacteria may differ considerably from that of other gram-negative bacteria, and PilP may have been replaced by other unidentified proteins. Probably for the same reason, pilotins like *Pseudomonas* PilF or *Neisseria* PilW and other accessory proteins, which are involved in the formation of the secretin pore in many gram-negative bacteria [35] have not been identified in cyanobacteria. Disruption of the pilus platform protein PilC abolished motility and natural competence [6]. Moreover both pili morphotypes (thin and thick pili) were absent from the cell surface of this mutant. 

### 3.2. The Secretion ATPases

The two ATPases PilB and PilT are required for pilus growth and its depolymerization, respectively. They belong to the type II/IV secretion ATPases [36] which are involved in protein transport across the outer membrane. The ATPases consist of an N-terminal and a C-terminal domain and assemble into hexameric ring structures [37,38,39]. Their function as molecular motors requires binding and hydrolysis of ATP. In case of PilB ATP hydrolysis leads to a conformational change of the protein, which is suggested to trigger the integration of a pilin subunit from the inner membrane into the growing pilus rod [39].

All cyanobacteria encoding *pil*-like genes harbor a homolog of each of the secretion ATPases (PilB1/PilT1), which are typically encoded upstream of the *pilC* gene in a conserved locus (Figure 1b). Furthermore, most cyanobacteria encode at least one copy of a second PilT homolog (PilT2), which is characterized by a proline rich N-terminal extension. Albeit not as frequent and with a far greater sequence variability than PilT2, additional PilB homologs (designated as PilB2 in *Synechocystis* 6803) can also be found in a variety of different cyanobacteria. Both *Synechocystis* 6803 and *Nostoc* *punctiforme* encode two copies of *pilT* and *pilB*-like genes, respectively. A *Synechocystis* 6803 *pilB1* mutant lost type IV pili, competency and motility, whereas inactivation of *pilB2* did not affect pilus biogenesis or motility [7]. In *Synechocystis* 6803 and *Nostoc* *punctiforme* *pilT1* mutants are non-motile but hyperpiliated. This phenotype is consistent with the supposed function of PilT in pilus retraction [6,23]. Moreover PilT1 from *Synechocystis* 6803 and *Microcystis aeruginosa* show ATPase activity *in vitro* and the *Microcystis* *pilT1* complements the *pilT* mutant of *Pseudomonas aeruginosa* (but interestingly not that of *Synechocystis* 6803) [40,41]. This suggests that PilB1 and PilT1 constitute the essential motor proteins. *PilT2* mutants show negative phototaxis in *Synechocystis* 6803 [6] and a slightly reduced number of pili and infection frequency in *Nostoc punctiforme* [23]. Therefore, PilT2 is thought to be involved in the regulation of pilus function. Whether it exerts its function as a homomer or a heteromultimer composed of PilT1 and PilT2 subunits is not known. The function of the additional PilB homologs in cyanobacteria has not been elucidated.

We hypothesize that additional proteins are associated with the secretion ATPases in many cyanobacteria and regulate their activity (Figure 1a). Cyanobacterial PilB1 homologs (with the exception of some *Prochlorococcus* species) are characterized by a highly conserved C-terminal domain with an invariant tetra cysteine motif resembling a zinc finger. This domain is lacking in PilB proteins from other bacteria and in cyanobacterial PilB2 homologs. In *Synechocystis* 6803, this specific C-terminal domain of PilB1 interacts with Hfq and is responsible for the correct localization and function of the RNA-chaperone which in turn is essential for biogenesis of both thick and thin pili [42,43]. Considering the co-occurrence of *hfq* and *pilB1* genes in cyanobacterial genomes we speculate that this interaction may be conserved among different species. Another candidate is the *Synechocystis* 6803 *slr0845* gene and its homologs, which are specific for clade B cyanobacteria [44]. They show a high degree of synteny and are predominantly located directly downstream of the *pilB/T/C* gene locus. In *Synechocystis* 6803 we could co-purify Slr0845 together with Hfq and PilB1 (unpublished) and a protein interaction study demonstrated an interaction between Slr0845 and PilL (TaxAY3) a histidine kinase involved in the regulation of motility [45,46].

### 3.3. The Pilus Rod

The pilus is composed predominantly of a major structural subunit, the so-called major pilin, and contains additional minor pilin subunits that may be required for assembly or specific pilus functions. Pilin proteins as well as the homologous pseudopilins of type II secretion systems are synthesized as prepilin precursors, which are characterized (and easily identified) by a distinct N-terminal signal peptide. During maturation the prepilin peptidase PilD removes the leader peptide and methylates the N-terminal amino acid of the mature protein [47]. Both pili morphotypes are absent on the cell surface of a *Synechocystis* 6803 *pilD* mutant which consequently is non-motile and lost transformation competency [6,7]. Interestingly, Linhartová *et al.* [47] showed that a *pilD* mutant in the non-motile *Synechocystis* 6803 wild-type background (the so called glucose-tolerant strain [48]) did not grow photoautotrophically because of a defect in the synthesis of photosystem II subunits. Most probably the unprocessed pilins interfere with translocation of photosystem II membrane subunits. Inactivation of *pilD* in *Nostoc* *punctiforme* led to non-motile mutant cells. However, *pilD* mutants were still able to assemble shortened pili. In addition, infection efficiency and symbiotic growth were severely attenuated in this mutant. [23]. From the gene products of eleven putative prepilin genes (*pilA1-pilA11*) identified in *Synechocystis* 6803, PilA1 constitutes the major pilin and is critical for type IV pili biogenesis, motility and transformation [5]. While the pilins PilA2*–*PilA8 seem to be dispensable for pilus biogenesis and motility [6,7], *pilA9–pilA11* mutants show a non-motile phenotype but retain normal type IV pili on the cell surface [49,50]. The PilA3 protein can be disregarded as a pilin since it shows no clear conservation of the signal peptide processing site and was recently characterized as a TatA homolog [51]. There are no conclusive data about the composition of pili in other cyanobacteria. Mutagenesis studies on prepilin genes have been further performed only for *Nostoc punctiforme*, which contains five (pseudo*-*)pilin-like genes. Duggan *et al.* [23] inactivated three of these genes, but only one mutant (in the locus NpF0069) was successfully segregated. This *pilA* mutant showed normal pilus biogenesis in hormogonia, although its infection efficiency was attenuated [23]. In *Synechocystis* 6803 post-translational modification of pilin subunits is thought to be critical for the biogenesis of functional pili. This includes O-linked glycosylation of pilin subunits and trimethylation of pilin at the C-terminal lysine [52,53,54]. 

### 3.4. Distribution of Pili Genes in Cyanobacterial Genomes

A systematic search using NCBI PSI-BLAST with default parameters revealed that type IV pilus biogenesis genes are conserved in almost all cyanobacterial species from all the major clades, including the phylogenetic distant *Gloeobacter* lineage (Figure 1b). While the number and organization of *pilA* genes coding for prepilins is variable, the organization of other core genes is conserved among cyanobacteria. The *pilMNOQ* genes encoding the outer membrane pore and the alignment complex as well as genes encoding the inner platform protein PilC together with the essential secretion ATPase genes *pilB1* and *pilT1* are generally clustered in the same order in two distinct loci. This implies that pili genes in cyanobacteria are phylogenetically ancient and were already present in a common ancestor. Remarkably, a complete set of these core genes is conserved in many species for which type IV pili (or other cell appendages) have never been described. Moreover, these species are not known to exhibit twitching motility or natural competence. Hence, it should be considered that at least in some cyanobacteria the type IV pilus apparatus has a function unrelated to motility or DNA uptake. Regarding the homology to type II secretion systems, a role in macromolecule secretion via a pseudopilus seems likely as discussed in the next paragraph. 

## 4. Function of Type IV Pili

Already more than 30 years have passed since the motility of certain filamentous cyanobacteria was analyzed in detail [55], however the mechanism of gliding motility of these cyanobacteria on a solid surface was not well defined. It was proposed that motility in *Phormidium unicinatum* and possibly in several other filamentous cyanobacteria is based on a junctional pore complex for directional extrusion of slime [25]. In addition, there is also evidence suggesting that arrays of contractile fibrils function in generating thrust for gliding motility [56]. However, several studies demonstrate the involvement of type IV pili in movement of filamentous cyanobacteria. Hormogonia, which are shorter filaments involved in dispersal and symbiotic associations with plants and fungi, are peritrichously piliated [23], suggesting that type IV pili are involved at least in hormogonial gliding. The core genes required for biogenesis of type IV pili are encoded in the genome of *Nostoc punctiforme* and *pilT* mutants are non-motile [23]. Risser *et al.* [24] used immunofluorescent staining to detect PilA on the surface of hormogonia. They demonstrated a fluorescence signal localized to a ring at the junction between individual cells showing a bias to one cell pole that is consistent among the cells of a particular hormogonium. Non-motile strains did not show a directional bias of PilA localization to one side of the septum, in contrast to motile strains. The authors hypothesize that the putative PilA protein they have analyzed might be part of the junctional pore complex. Thus, it is possible that type IV pili in hormogonia are mainly involved in secretion of polysaccharides. Although there is considerable evidence that polysaccharide export is important for motility in *Nostoc* *punctiforme* [57] this is no clear evidence for polysaccharide “jet propulsion”: The requirement may simply be to provide a suitable surface for gliding, as appears to be the case in *Synechocystis* 6803 or *Myxococcus* *xanthus* [58,59].

Apart from their importance in motility type IV pili are essential for natural competence of *Synechocystis* 6803. Inactivation of genes encoding components of the pilus apparatus inhibit the uptake of exogenous DNA [7] suggesting that type IV pili are part of the DNA-uptake machinery in *Synechocystis* 6803. Most DNA-uptake complexes of gram-negative naturally competent bacteria include a type IV-like pilus as a core component. Based on data obtained with *Vibrio cholera* Seitz and Blokesch [60] suggest a two-step process for uptake of exogenous DNA, where the type IV pili are responsible for the translocation of incoming DNA across the outer membrane. Several competency proteins in a pilus-independent process then facilitate further transport into the cytoplasm.

In addition, there is evidence that pili function as nanowires facilitating electron transfer to extracellular electron acceptors [61]. Lamb *et al.* [62] published data on *Synechocystis* 6803 showing that a *pilA1* mutant grows slower than the wild type on oxidized iron minerals. They speculate that the PilA1 protein may be important for transport of electrons to these iron oxides. However, in this study a non-motile strain harboring a mutation in *pilC* was used. The importance of an intact type IV pili structure and function for the transport of electrons has still to be analyzed. 

A new function of type IV pili has been demonstrated in our lab [43]. The RNA chaperone Hfq facilitates the action of small non-coding regulatory RNA molecules (sRNA) in many bacteria [63]. *Synechocystis* 6803 encodes several sRNAs but involvement of Hfq in the function of these RNA regulators has not been shown so far, though the expression of some mRNAs and sRNAs was altered in an *hfq* mutant. Inactivation of *hfq* in *Synechocystis* 6803 leads to a phenotype: Mutant cells have lost all detectable appendages on their surface and are non-motile and non-transformable [42]. Surprisingly, Hfq binds to the pilus base via the C-terminal part of the PilB1 protein, which is unique in cyanobacterial PilB proteins. Whereas Hfq seems to be important for type IV pili assembly, pili are also important for Hfq function. Expression of Hfq-dependent transcripts is altered when *pilB1* or *pilC* are inactivated and when Hfq is not correctly localized to the pilus base due to different single amino acid changes in the PilB1 binding sites of Hfq. Consequently, all *Synechocystis* 6803 strains with mutations in pili genes or which are non-motile due to mutations in regulatory components might also be defective in Hfq function.

The composition of the type IV pili apparatus shares important similarities to type II secretion systems [64]. Secretion of proteins, which are not related to pilins was shown to be type-IV-pili-dependent in a range of bacteria (e.g., Kirn *et al.* [65]). Most cyanobacteria harbor genes that are similar to *pilB* and other genes for components of the type IV pili apparatus, but do not assemble pili on their surface [66,67]. The role of these gene products is unclear, however a function in protein secretion might be possible. Schatz *et al.* [68] showed that biofilm formation of *Synechococcus elongatus* PCC 7942 cells is regulated by an extracellular factor. This factor is most probably not secreted anymore to the medium in *pilB* and *pilC* mutants. Assuming that type IV pili are also involved in *Synechocystis* 6803 protein secretion, deletion of *hfq* might affect protein secretion thereby having secondary effects on putative extracellular signals.

## 5. Pili Assembled by the Chaperone-Usher Pathway (CU pili)

**Figure 2:**
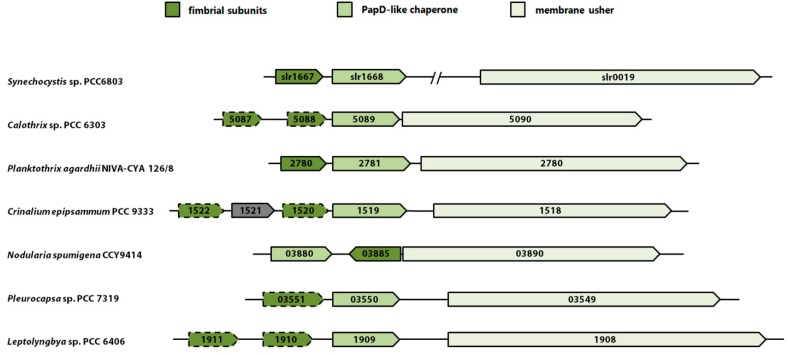
Synteny of gene clusters encoding all the major components of putative CU pili in different cyanobacteria compared to *Synechocystis* 6803 where the usher homolog is located elsewhere in the genome. All putative CU pili related cyanobacterial gene clusters exhibiting a conserved domain structure (putative subunits PFAM05229; chaperone PFAM00345; usher PFAM00577) are shown. Weak homology is indicated by broken lines.

Yoshimura *et al.* [69] suggested that two proteins encoded by the genes *slr1667* and *slr1668* (named construction of cell surface components—*cccS* and *cccP*, respectively) are involved in biogenesis of the thick pili in *Synechocystis* 6803. Immunocytochemical analysis supported the idea that CccS was localized at the cell surface region and CccP in the cell periplasm. Both *cccS* and *cccP* mutants are non-motile and have no or less thick pili on their surface as shown by electron microscopy. Interestingly, *cccP* and *cccS* transcripts are the most differentially accumulated mRNAs in *sycrp1* as well as *hfq* mutants, which are also non-motile. In their paper Yoshimura *et al.* [69] proposed a model, where CccS and/or CccP support targeting or stable assembly of the PilQ channel, thereby affecting assembly of thick pili. In addition, they state, that thin pili are not affected, suggesting that CccP and CccS are not involved in assembly of these structures. However, a different hypothesis can be drawn from the analysis of the amino acid sequences of both proteins. CccS exhibits a spore coat protein U-domain (SCPU, PFAM05229), whereas CccP contains a PapD-N domain (PFAM00345) [69]. These domains are typically found in proteins involved in biogenesis of CU pili in gram-negative bacteria via the chaperon/usher pathway. Usually, the respective genes are organized in operons containing at least one subunit of the fimbriae, a chaperone and a membrane protein for translocation of the subunits, the so called usher [18]. During assembly of the CU pili the signal peptide of a fimbriae subunit is cleaved off during its translocation across the cytoplasmic membrane. Further, the chaperone protein assists the correct folding and stabilization of the subunit. After binding to the usher protein, which forms a pore in the outer membrane, the chaperone is displaced and the fimbriae subunit is integrated into a filament on the cell surface [70]. In accordance with the domain structure, CccS might represent a subunit of the CU pilus and CccP the corresponding chaperone. It was already shown that CccS has a signal peptide, which is cleaved off, and that CccS is transported across the outer membrane in a CccP-dependent way [69]. These authors did not link CccSP with assembly of CU pili-like structures on the surface of *Synechocystis* 6803, most probably because there is no usher homolog encoded in this operon. However, in a phylogenetic analysis Nuccio and Bäumler [18] identified an usher homolog (slr0019, PFAM00577) in *Synechocystis* 6803. A search in the JGI IMG database (http://img.jgi.doe.gov) for proteins containing usher domains revealed the existence of further *slr0019* homologs in different cyanobacteria. Remarkably, in most cases these genes are located directly downstream of a putative *cccSP* operon and thus represent the classical chaperone/usher operon structure (Figure 2). Thus, it seems plausible that CccSP together with Slr0019 could form CU-like pilus structures defined as the thin pili in *Synechocystis* 6803 by electron microscopy analysis. Further, their incorrect assembly may affect biogenesis of thick pili, indirectly. This hypothesis is contradictory to the data shown by Yoshimura *et al.* [69], which suggest that formation of thin pili was not affected in *cccS* and *cccP* mutants. However, in this paper it was also mentioned that, whereas bundles of thin-pili-like structures were clearly visualized by negative straining, this was not possible for thin pili. As the subunit composition of these different structures is not known so far, it is hardly possible to conclude from electron microscopy data about the nature of the various pili structures. Clearly, a comprehensive inspection of surface structures, including analysis of an *slr0019* mutant, is needed to link putative CU pili related gene products to the respective surface appendages.

## 6. Conclusions

Many cyanobacteria harbor genes for the assembly of pili, but their function is largely unknown apart from one model strain. Functionally, non-flagellar appendages should have important roles in biofilm formation, aggregation, adhesion, natural competence or secretion, also in non-motile cyanobacteria. In addition, cyanobacterial appendages might have specialized roles and specific implications for these phototrophic organisms, which are different from other non-phototrophic bacteria. A different mechanism of Hfq function as well as effects of mutations in pili genes on photosynthetic functions might be only the beginning of the understanding how appendages are involved in cyanobacterial growth. In this respect it is important to note that various cyanobacterial wild-type strains are used in different laboratories all over the world. At least for *Synechocystis* 6803, the most important model strain for the study of motility and the function of pili, many laboratories work with the non-motile GT-strain, which was sequenced in 1996 [71]. This strain harbors several mutations, including a frame shift mutation in *pilC* and a mutation in the serine threonine kinase *spkA*, which is also involved in motility [72]. Over the few last years, motile sub-strains of this species have been re-sequenced [73,74] revealing even more differences between the strains. For this reason, physiological studies or microarray analyses in laboratories using different strains of *Synechocystis* 6803 may give conflicting results. Because there are so many genes involved in pilus assembly and regulation of motility mutations occur very often. There is also a bias for picking non-motile strains, especially because these colonies appear to be nicely shaped. It should be stressed here that known differences in genome sequences have to be considered when planning certain experiments and when comparing mutant strains from different wild-type backgrounds even in the same laboratory.
